# Functional differences in scavenger communities and the speed of carcass decomposition

**DOI:** 10.1002/ece3.8576

**Published:** 2022-02-22

**Authors:** Elke Wenting, Salomé C. Y. Rinzema, Frank van Langevelde

**Affiliations:** ^1^ Department of Environmental Sciences Wageningen University and Research Wageningen The Netherlands; ^2^ 6029 Department of Animal Ecology and Physiology Institute for Water and Wetland Research Radboud University Nijmegen The Netherlands

**Keywords:** carcass decomposition, scavenger community, vertebrate scavengers

## Abstract

Carcass decomposition largely depends on vertebrate scavengers. However, how behavioral differences between vertebrate scavenger species, the dominance of certain species, and the diversity of the vertebrate scavenger community affect the speed of carcass decomposition is poorly understood. As scavenging is an overlooked trophic interaction, studying the different functional roles of vertebrate species in the scavenging process increases our understanding about the effect of the vertebrate scavenger community on carcass decomposition. We used motion‐triggered infrared camera trap footages to profile the behavior and activity of vertebrate scavengers visiting carcasses in Dutch nature areas. We grouped vertebrate scavengers with similar functional roles. We found a clear distinction between occasional scavengers and more specialized scavengers, and we found wild boar (*Sus scrofa*) to be the dominant scavenger species in our study system. We showed that these groups are functionally different within the scavenger community. We found that overall vertebrate scavenger diversity was positively correlated with carcass decomposition speed. With these findings, our study contributes to the understanding about the different functional roles scavengers can have in ecological communities.

## Graphical abstract

We examined functional differences within the scavenger community and found groups of scavengers with similar roles. Wild boar (*Sus scrofa*) was determined to be the dominant scavenger in our system, accelerating the carcass decomposition speed.

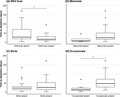

## 1 INTRODUCTION

The decomposition of organic matter (i.e., detritus) is a crucial part of the cycling of energy and nutrients in all ecosystems (Moore et al., 2004; Swift et al., 1979). Up to 90% of the detritus pool consists of plant matter, which represents an enormous quantity in comparison with animal detritus (Barton et al., 2013; Swift et al., 1979). Despite being such a disproportionally small part of the overall detritus pool, animal detritus, predominantly in the form of carcasses, has been shown to largely affect ecological communities (e.g., Barton et al., 2013; Barton et al., 2019; Benbow et al., 2019), for instance as nutrient‐rich food source for many organisms (Schoenly & Reid, 1983), through its role in the nutrient cycle (e.g., Benbow et al., 2019; Parmenter & MacMahon, 2009), and by stabilizing food webs (Wilson & Wolkovich, 2011).

The large impact that carcasses may have on ecosystem functioning can be attributed to the ubiquitous nature of the scavenging behavior across species (DeVault et al., 2003; Wilson & Wolkovich, 2011). We use the term “scavengers” for all animal species that are involved in the process of carcass decomposition. Although scavenging is often overlooked in assessments of vertebrate diet composition (DeVault et al., 2003), vertebrate scavengers are often the primary consumers of carcasses in terrestrial ecosystems, consuming 35% to 75% of the total carrion pool (DeVault et al., 2003).

The majority of scavenger species consumes carcasses facultatively, meaning that carcasses occupy varying degrees of importance in these species’ diet in addition to other food sources and that these species could survive without it (DeVault et al., 2003; Pereira et al., 2014; Wilson & Wolkovich, 2011). Although facultative scavenging is often considered to be an opportunistic feeding mechanism, it has recently been shown to be a highly regulated and constant behavior for most species, governed by a variety of intrinsic and external factors (Selva et al., 2005), such as carcass type (Olson et al., 2016), habitat characteristics (Smith et al., 2017), and interguild and intraguild interactions (Inagaki et al., 2020; Selva & Fortuna, 2007).

Different scavenger species may fulfill different functional roles in the carcass decomposition process (Sebastián‐González et al., 2020, 2021), which would be reflected in differences in behavior and preferences for tissues types. For example, Young et al. (2014) observed that common buzzards (*Buteo buteo*) fed primarily on soft tissues in the early stages of decomposition, while carrion crows (*Corvus corone*) increased their feeding as carcasses went into later stages of decomposition and exploited more different body parts. Due to such functional differences, the vertebrate species in the scavenger guild may complement each other in the removal of carcasses (Olson et al., 2016). Cortés‐Avizanda et al. (2012), for instance, suggested that a diverse and species‐rich scavenger community that functions synergistically may be the key to the stability and efficacy of carcass removal as an ecosystem service. Olson et al. (2012) found that the exclusion of an important scavenger species from the scavenger guild resulted in incomplete carrion depletion, even when the remaining species exhibited a compensatory response to the reduced competition. Accordingly, Hill et al. (2018) found that the exclusion of vultures resulted in decreased scavenging by facultative scavengers and overall slower decomposition rates. Additionally, Selva and Fortuna (2007) found that rare scavenger species were more likely to forage on carcasses that had already been frequently visited by more common scavengers, and suggested that these rare species matched their carcass choice with that of scavenger specialists such as the common raven (*Corvus corax*).

However, the different functional roles of vertebrate scavenger species and how these relate to the speed of carcass decomposition are still poorly understood (Barton et al., 2013). As scavenging is a frequently overlooked trophic interaction, studying the different functional roles of vertebrate species in the scavenging process increases our understanding of the effect of the vertebrate scavenger community on carcass decomposition. This study aimed to determine the different functional roles of the vertebrate scavengers, and how the diversity within the scavenger community relates to carcass decomposition speed. We predicted that higher vertebrate scavenger diversity would result in more efficient carcass exploitation and therefore in faster carcass decomposition (Griffin et al., 2008; Hooper et al., 2005).

## 2 METHODS

### 2.1 Study system

**FIGURE 1:**
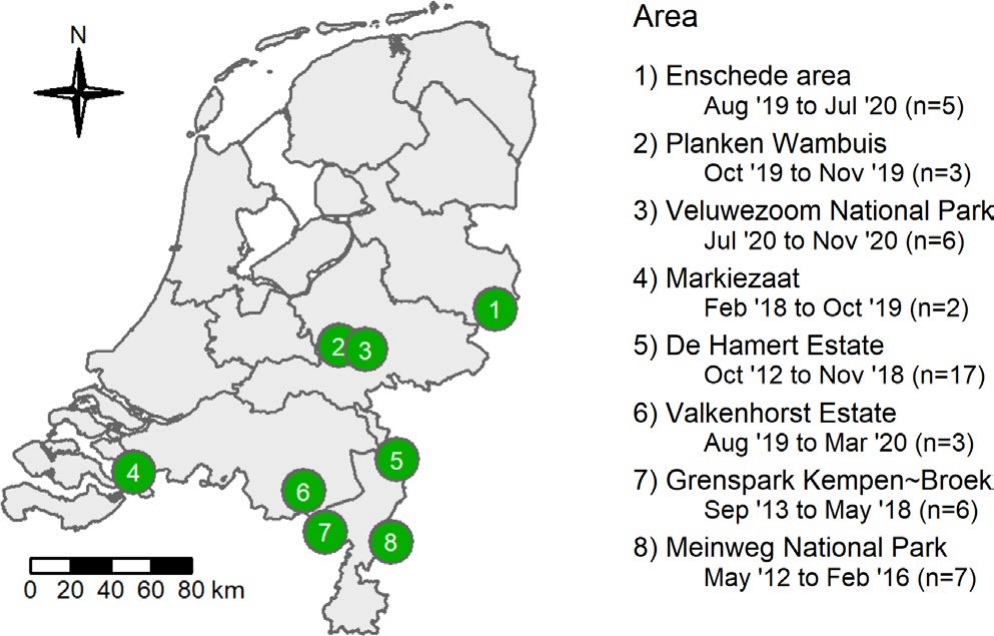
Map of the Netherlands showing the areas where we monitored carcasses until depletion. The period of monitoring and the number of monitored carcasses per area are indicated

We monitored the vertebrate animals that visited 49 carcasses in eight Dutch nature areas, in different periods between May 2012 and November 2020 (Figure 1). A minimum of two carcasses had been monitored in each of these areas. The carcasses were placed in heathlands or forested areas, whereby we avoided completely open or densely forested places. Different vertebrate scavenger communities were present in these areas, allowing us to study the effect of different scavenger guilds on the carcass decomposition speed.

### 2.2 Field methods

We used motion‐triggered infrared camera traps to monitor the decomposition process of carcasses. Different models of camera traps were used throughout the years but all were part of the Bushnell Trophy Cam product line. We attached the camera traps to trees, shrubs, or actively placed poles at a distance of two meters from the carcass at one‐meter height and slightly bent forward pointing toward the ground, depending on the local circumstances. All carcasses were positioned with the abdomen or back to the camera, and tied by the front and rear legs to trees or poles using natural ropes to prevent the carcasses from getting dragged out of view. The camera traps were set to videos of 60 seconds per trigger, with a two‐ or three‐second interval between the triggers depending on the exact camera model. We visited the carcasses approximately every two weeks to replace the 32 or 64 GB SD card and to renew the batteries. We minimized the time spent and the number of people present at the carcass site as much as possible to reduce possible anthropogenic disturbance.

In our analyses, we only included carcasses of which the whole decomposition process was monitored, resulting in a total of 49 carcasses (Table S1). The carcasses were obtained from roadkills, except for Planken Wambuis and Veluwezoom National Park, where the carcasses were obtained from culling. No animals were killed for the purpose of this study. In total, we monitored the carcasses of 33 roe deer (*Capreolus capreolus*), seven wild boar (*Sus scrofa*), four European badger (*Meles meles*), three red deer (*Cervus elaphus*), one sheep (*Ovis orientalis*), and one fallow deer (*Dama dama*). Only complete carcasses were monitored, that is, no guts only.

### 2.3 Annotation camera trapping videos

The collected camera trapping videos were uploaded to the online platform Agouti (WUR & INBO, 2021), from which the footage was annotated. Per video, we annotated (1) the species and the number of individuals; (2) the behavior of these animals (Table 1); (3) if applicable, the tissues that were eaten or collected; and (4) the stage of decomposition of the carcass. For the behavior and tissues, we annotated the longest and second‐longest shown behavior or eaten tissue type, resulting in a maximum of two observations each. For simplicity, we did not distinguish between these in the further analyses, meaning that both the longest and second‐longest shown behaviors or eaten tissue type were treated equally. In the case that two or more species visited the carcass in the same video, we annotated the video for each species separately.

**TABLE 1:** Overview of the definitions we used to annotate the behavior of the scavengers that were recorded by the camera traps

Behavior	Abbreviation	Definition
Passing	PAS	Move in front of camera trap without moving body and/or head in the direction of the carcass.
Interest	INT	Body and/or head moves toward the carcass, or mouth/beak touches the carcass without any chewing/picking movements.
Eating	EAT	Mouth/beak touches the carcass, and removing carcass parts by chewing/picking movements.
Standing on carcass	STA	Touching the carcass with legs only; that is, no other body parts other than legs touch the carcass.
Intraspecific interaction	INTRA	Physical and non‐physical contact between individuals of the same species.
Interspecific interaction	INTER	Physical and non‐physical contact between individuals of a different species.
Collecting material	CM	Taking along carcass parts in the direct vicinity of the carcass.

Based on the quality of the videos, we were able to distinguish between seven tissue types and three stages of decomposition. For the tissue types, we distinguished between: (1) bones and hooves (hereafter “bones”); (2) hairs; (3) nose, ears, eyes, anus, and skin on the armpits and abdominal region (hereafter “soft tissues”); (4) skin on other parts of the body (hereafter “skin”); (5) muscle; (6) organs; and (7) insects and larvae that were present on the carcass (hereafter “insects”), that is, indirect carcass consumption. Some behavioral and tissue observations were annotated as unknown. We excluded these observations from the analysis.

For the stages of decomposition, we distinguished between: (1) the bloated stage, in which the carcass is fresh and/or abdominal bloating occurs due to anaerobic microbial activity, and the carcass has no or only minor injuries that do not expose any entrails; (2) the active decay stage, characterized by rapid mass and volume loss due to increased scavenger activity, and during which at least some entrails are exposed; and (3) the advanced decay stage, characterized by a flat abdomen and only some parts of the skin and skeleton remains, possibly supplemented by some other tissue leftovers (Feddern et al., 2019). Twelve carcasses had such major injuries due to the cause of death that their decomposition started in the active decay stage.

Per carcass, we noted the day the carcass was placed and the day the carcass was fully decomposed to calculate the time to depletion per carcass. The carcass was considered as fully decomposed at the end of the advanced decay stage, when none of the carcass remains were visible anymore. The average daily temperature, based on the mean daily temperature from the nearest weather station (KNMI, 2021), was calculated to include in the further analyses since the ambient temperature has been shown to be a primary determinant of carcass longevity (e.g., Farwig et al., 2014; Parmenter & MacMahon, 2009; Ray et al., 2014). We also noted the start month, with January 2012 as month 1—since the first carcasses were monitored in 2012—to correct for temporal autocorrelation.

## 3 STATISTICAL ANALYSES AND RESULTS

### 3.1 Functional scavenger groups

All statistical analyses were done in R version 4.0.2 (R Core Team, 2020). In total, we annotated 6805 videos of vertebrates visiting the carcasses. Below, we discuss the statistical analyses together with the results as further analyses were determined based on the foregoing results.

We started by determining the functional groups of scavengers in five steps. First, we selected the species that we included in the further analyses. This was done by selecting the species that showed eating behavior, and from these species, we only selected the species with at least 30 observations. This resulted in a total of 17 species: beech marten (*Martes foina*), carrion crow, cattle (*Bos taurus*), common buzzard, common raven, domestic cat (*Felis catus*), domestic dog (*Canis lupus familiaris*), European polecat (*Mustela putorius*), fieldfare (*Turdus pilaris*), great tit (*Parus major*), horse (*Equus caballus*), mistle thrush (*Turdus viscivorus*), red fox (*Vulpes vulpes*), roe deer, song thrush (*Turdus philomelos*), wild boar, and wood mouse (*Apodemus sylvaticus*). After excluding the videos of the other species from the data, there were 6548 videos left. In total, we had 9100 observations of behavioral types and 6752 observations of tissue types.

Second, for each species, we calculated the percentage of observations per decomposition stage, per behavioral type, and per tissue type (Table 2).

**TABLE 2:** Percentage of observations per decomposition stage, behavior, and tissue type, per selected species

	Bloated stage	Active decay	Advanced decay	CM	EAT	INT	INTER	INTRA	PAS	STA	Bones	Hairs	Insects	Muscle	Organs	Skin	Soft	Detection time	Time till scavenging	Adult body mass
*B. taurus*	51.6	26.6	21.9	0.0	4.8	27.4	0.0	1.2	66.7	0.0	50.0	0.0	0.0	0.0	0.0	50.0	0.0	5550	836	613,000
*E. caballus*	44.1	54.4	1.5	0.0	5.2	46.3	0.0	16.4	32.1	0.0	0.0	0.0	0.0	0.0	0.0	37.5	62.5	3	50	400,000
*A. sylvaticus*	1.0	0.0	99.0	2.0	2.0	27.7	0.0	0.0	68.3	0.0	0.0	0.0	0.0	0.0	0.0	0.0	0.0	35,450	45,552	21.9
*C. capreolus*	8.5	22.0	69.5	0.0	3.0	26.2	0.0	0.0	70.8	0.0	0.0	50.0	0.0	0.0	0.0	50.0	0.0	36,970	28,054	22,502
*P. major*	33.3	36.7	30.0	28.6	11.4	11.4	0.0	0.0	48.6	0.0	0.0	90.91	9.1	0.0	0.0	0.0	0.0	48,780	41,098	19.25
*T. philomelos*	3.1	3.1	93.8	2.7	19.9	5.4	0.0	0.0	62.2	10.8	0.0	20.0	80.0	0.0	0.0	0.0	0.0	35,055	36,148	67.75
*T. pilaris*	0.0	0.0	100	0.0	30.2	0.0	0.0	0.0	69.8	0.0	0.0	0.0	100	0.0	0.0	0.0	0.0	14,348	17,311	106
*T. viscivorus*	0.0	0.0	100	0.0	12.9	0.0	0.0	3.2	83.9	0.0	0.0	0.0	0.0	0.0	0.0	0.0	0.0	11,291	14,066	117.50
*B. buteo*	8.6	81.9	9.5	0.2	65.1	12.2	3.2	2.1	4.2	13.1	0.8	1.31	0.0	37.1	12.2	13.5	35.2	17,896	12,631	875
*C. corax*	1.6	45.9	52.5	4.5	67.9	3.6	0.9	16.1	4.7	2.4	2.4	4.67	0.3	44.7	17.7	21.3	9.1	7369	7514	1200
*C. corone*	8.4	86.2	5.4	1.2	37.7	27.2	6.0	4.3	22.1	1.5	1.5	4.04	3.5	59.1	3.5	6.6	21.7	12,427	12,319	375
*C. lupus familias*	7.0	7.0	86.0	0.0	47.7	30.8	1.5	1.5	19.5	0.0	14.3	0.0	0.0	4.8	4.8	76.2	0.0	23,727	15,217	35,000
*F. catus*	18.0	63.9	18.0	0.0	47.1	31.0	1.2	0.0	20.7	0.0	0.0	0.0	0.0	53.1	0.0	43.8	3.1	34,149	6764	2885
*M. foina*	10.6	32.9	56.5	1.7	20.7	43.8	1.7	0.8	31.4	0.0	62.5	12.50	0.0	0.0	0.0	25.0	0.0	30,607	20,530	1675
*M. putorius*	13.3	32.7	54.0	1.4	59.8	27.7	1.0	0.6	11.3	0.2	0.5	1.68	0.0	42.5	2.9	42.0	10.6	46,149	15,178	975.55
*V. vulpes*	8.9	40.6	50.5	4.6	41.7	30.5	0.7	0.5	22.0	0.0	21.4	2.88	2.1	21.4	9.9	33.9	8.4	18,922	25,656	4820
*S. scrofa*	6.8	33.5	59.7	0.8	66.2	14.6	0.7	7.6	10.2	0.0	5.6	1.46	0.2	49.1	7.6	33.1	2.9	8706	19,934	84,471

Third, we calculated for each species the average detection time—that is, time until a species visited a carcass for the first time—and the average time until first scavenging event—that is, time until first annotation of EAT or CM behavior (Table 2). Furthermore, we included the average adult body mass in grams for each species (Table 2; Jones et al., 2009), as a proxy of their capacity to tear open the carcass’ skin, exposing more body parts of the carcass, thus enabling more carcass parts being consumed (e.g., Freeman & Lemen, 2008).

**FIGURE 2:**
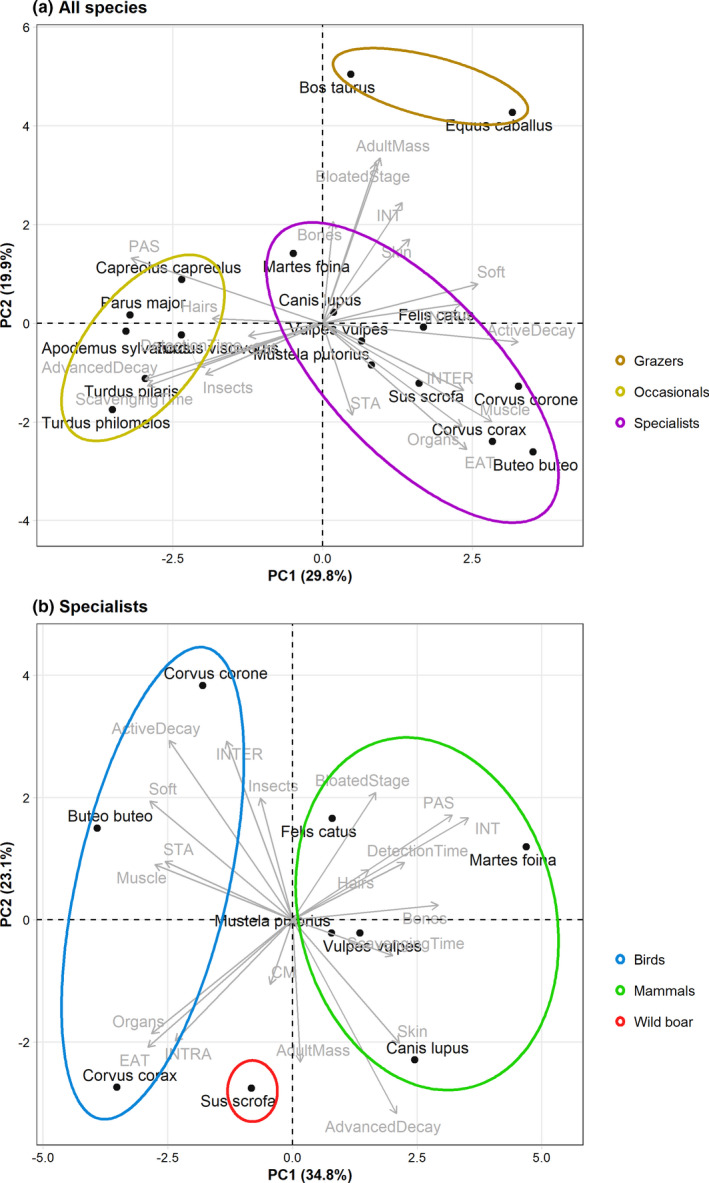
PCA biplots of (a) all the selected 17 species, and (b) the species defined as specialists. The circles indicate the scavenger groups we defined

Fourth, the collected information per species as described in the second and third step (Table 2) was analyzed with a principal component analysis (PCA) in order to group the species with the most similar scavenging habits (Figure 2a). This resulted in three groups: (1) the Grazers (G), consisting of cattle and horse; (2) the Occasionals (O), consisting of fieldfare, great tit, mistle thrush, roe deer, song thrush, and wood mouse; and (3) the more specialized scavengers. Based on this PCA (Figure 2a), beech marten was located between the group Occasionals and Specialists, but due to its characteristics, especially low PAS behavior (Table 2), we decided to group this species with the Specialists. The group Grazers was characterized by a low percentage of EAT behavior (5% on average), and relatively high INT and PAS behavior (36.5% and 49% on average, respectively). This group can be characterized by its high adult body mass. The group Occasionals was characterized by relatively low EAT and CM behavior (12% and 5% on average, respectively) and a high percentage of PAS behavior (67% on average). The only tissue types identified for this group were hairs, insects, and skin.

Last, we used an additional PCA to further analyze the species we grouped as Specialists (Figure 2b). Based on this analysis, we subdivided this group into three groups: (1) the Birds (B), consisting of carrion crow, common buzzard, and common raven; (2) the Mammals (M), consisting of beech marten, domestic cat, domestic dog, European polecat, and red fox; and (3) the Wild boar (W). The group Birds was characterized by a prevalence for the active decay stage (71% on average) and the occurrence of STA behavior for all species. The group Mammals had a larger body mass on average than the group Birds (9,071 and 817 grams, respectively). Although species in the group Mammals were the most scattered and there was no single distinctive trait they all shared, this group was characterized by a percentage of PAS behavior lower than 35%. Due to its high body mass, high percentage of EAT behavior, and high percentage of INTRA behavior (Table 2), which indicates a larger group size compared with the other species, we decided to treat wild boar as a separate group. We used the FactoMineR (Le et al., 2008) and factoextra (Kassambara & Mundt, 2017) packages to compute and visualize both PCAs.

Summarized, for further analysis, we divided the scavengers that visited the carcasses into five groups: (1) the Grazers (G); (2) the Occasionals (O); (3) the Birds (B); (4) the Mammals (M); and (5) the Wild boar (W).

### 3.2 Carcass depletion time versus scavenger groups

We analyzed in three steps how the time until carcass depletion was influenced by the presence of particular scavenger groups. First, we made an overview of which groups were present per carcass. In total, there were 15 combinations of scavenger groups observed (Table S2a).

Second, since we noted that there were only six carcasses where the group Grazers was involved, spread over four combinations of groups, we tested whether the presence of the group Grazers influenced the depletion time, that is, time until the end of the advanced decay stage. We selected the carcasses with the combination of groups with the group Grazers present and the carcasses with the same combination of groups without the group Grazers present. Using a linear mixed‐effect model (Kuznetsova et al., 2017) with days to depletion as dependent variable, the group Grazers presence or absence and carcass initial state as fixed factors, mean daily temperature as covariate, and area, start month, and carcass species as random factors, we found no difference between the group Grazers presence or absence (LMM, *df* = 3.676, *F* = 0.388, *p* = .570). Carcass initial state (LMM, *df* = 17.975. *F* = 0.982, *p* = .335) and mean daily temperature (LMM, *df* = 16.445, *F* = 1.925, *p* = .184) were not significant. Therefore, we decided to constitute the carcass groups without incorporating the presence or absence of the group Grazers. This resulted in 11 combinations of groups (Table S2b).

**FIGURE 3:**
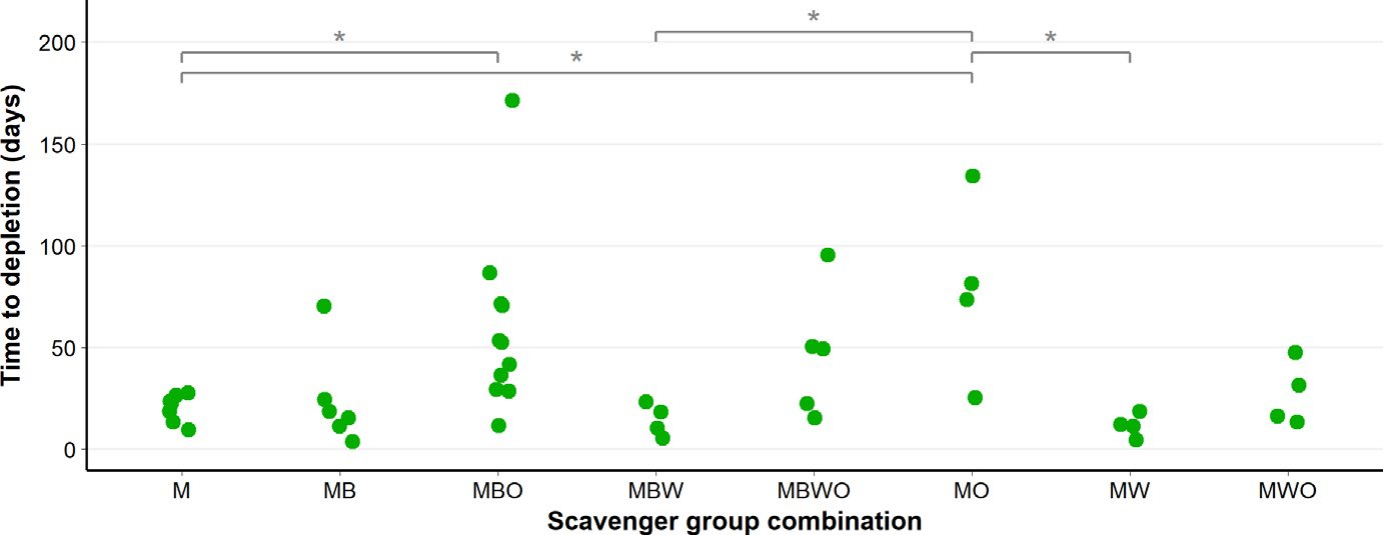
Time until carcass depletion per scavenger group combination. The scavenger group combinations from left to right: M, Mammals; MB, Mammals and Birds; MBO, Mammals, Birds, and Occasionals; MBW, Mammals, Birds, and Wild boar; MBWO, Mammals, Birds, Wild boar, and Occasionals; MO, Mammals and Occasionals; MW, Mammals and Wild Boar; and MWO, Mammals, Wild boar, and Occasionals. *p < .05

Last, from these 11 combinations of groups, we selected the combinations that represented at least four carcasses (Table S2c). This resulted in four carcasses being excluded from further analysis. In total, we analyzed eight combinations of groups for differences in depletion time (Figure 3), using a linear mixed‐effect model with days to depletion as dependent variable, the scavenger groups and carcass initial state as fixed factors, mean daily temperature as covariate, and area, start month, and carcass species as random factors. We found that carcasses with the groups Mammals and Occasionals present decomposed slower than three other groups: carcasses with only Mammals present; carcasses with Mammals, Birds, and Wild boar present; and carcasses with Mammals and Wild boar present. We also found that carcasses with only Mammals present decomposed faster than carcasses with Mammals, Birds, and Occasionals present (Figure 3; LMM, *df* = 22.673 *F* = 7.200; *p* < .001). Again, carcass initial state (LMM, *df* = 29.229, *F* = 3.957, *p* = .056) and mean daily temperature (LMM, *df* = 32.558, *F* = 2.295, *p* = .139) were not significant.

### 3.3 Dominant scavenger group

**FIGURE 4:**
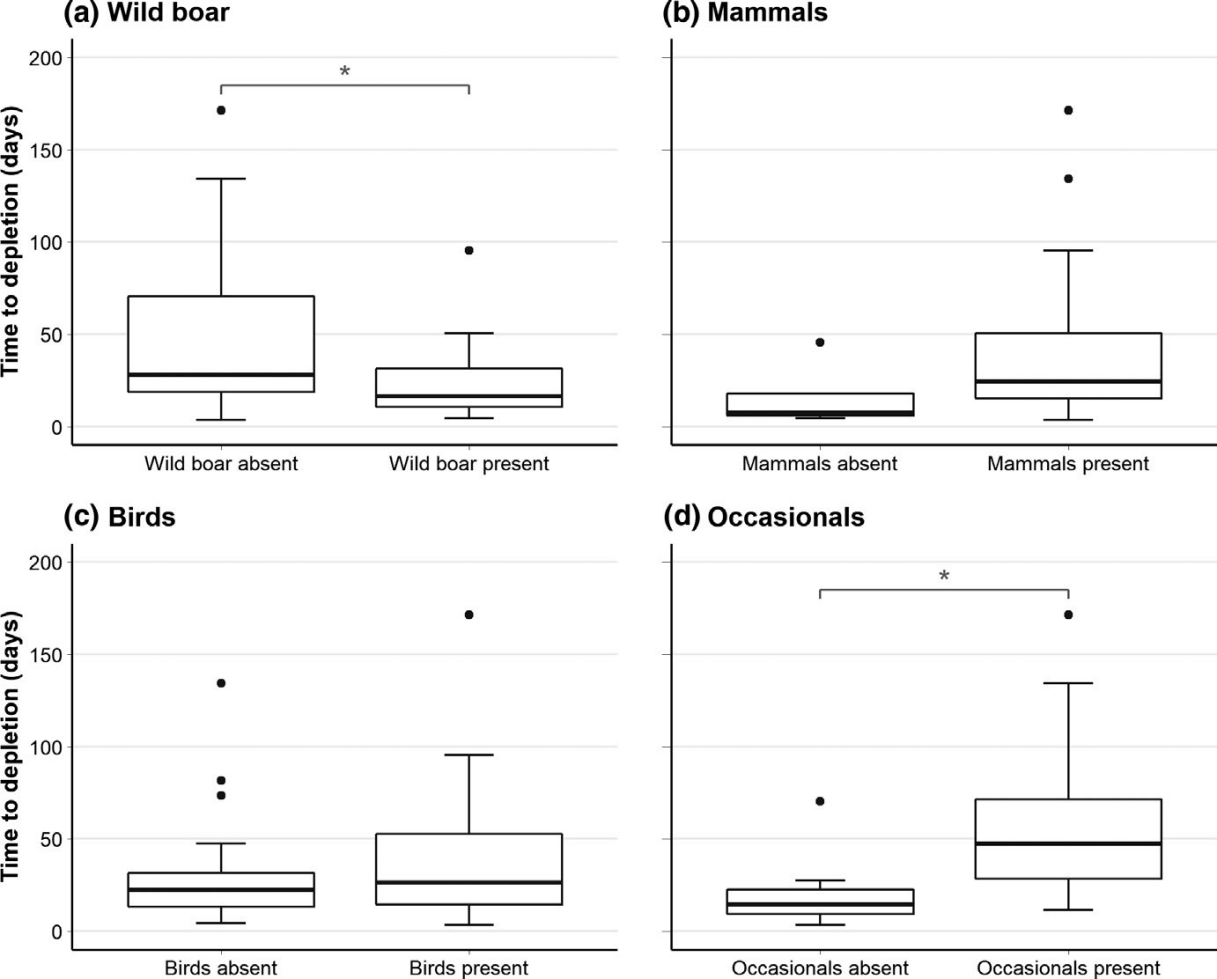
Time until carcass depletion per scavenger group presence: (a) the wild boar, (b) mammalian scavengers, (c) scavenging birds, and (d) occasionals. *p < .05

Next, we tested whether the decomposition process was significantly sped up by the presence of a particular scavenger group. We used again linear mixed‐effect models and included the same covariate, fixed factor, and random factors as described before. We found that the presence of the group Wild boar accelerated the time to carcass depletion (Figure 4a; LMM, *df* = 1, *F* = 4.509; *p* = .045). We did not find an effect for the presence of the group Mammals (Figure 4b; LMM, *df* = 1, *F* = 0.453, *p* = .504), nor for the presence of the group Birds (Figure 4c; LMM, *df* = 1, *F* = 1.035, *p* = .315). When the group Occasionals was present, we found that the time till depletion was longer than their absence (Figure 4d; LMM, *df* = 1, *F* = 14.373, *p* < .001). Thus, we defined the Wild boar as the dominant scavenger group in our study system.

### 3.4 Scavenger diversity

**FIGURE 5:**
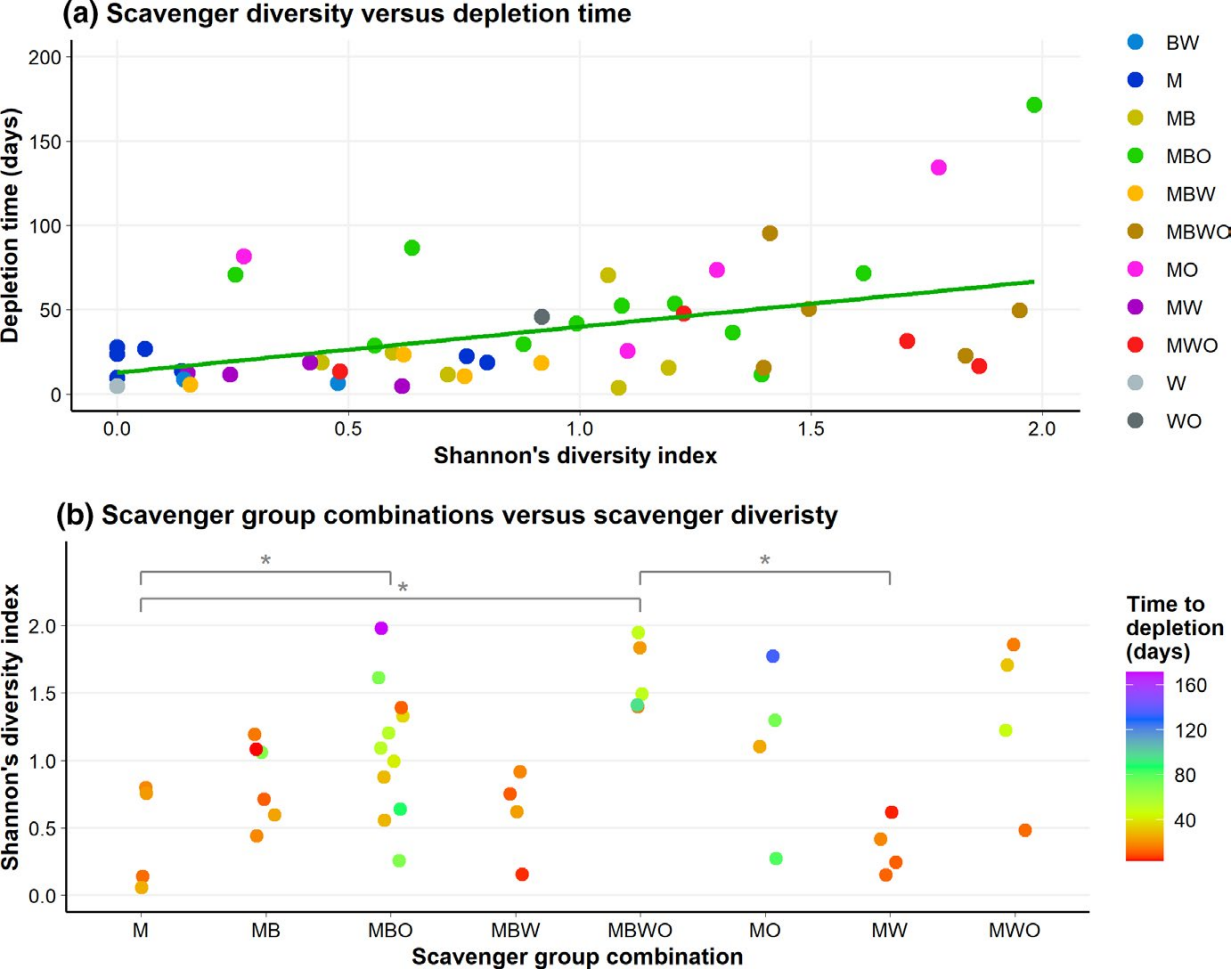
Scavenger diversity—as Shannon's diversity index—versus (a) the time until carcass depletion, and (b) over the scavenger group combinations. The scavenger group combinations: BW, Birds and Wild boar; M, Mammals; MB, Mammals and Birds; MBO, Mammals, Birds, and Occasionals; MBW, Mammals, Birds, and Wild boar; MBWO, Mammals, Birds, Wild boar, and Occasionals; MO, Mammals and Occasionals; MW, Mammals and Wild Boar; MWO, Mammals, Wild boar, and Occasionals; W, Wild boar; and WO, Wild boar and Occasionals. *p < .05

We studied the effect of scavenger diversity on the speed of carcass decomposition in two ways. First, we calculated Shannon's diversity index based on the vertebrate scavenger species per carcass (Oksanen et al., 2020). Using a linear mixed‐effect model with depletion time as dependent variable, diversity index and carcass initial state as fixed factors, mean daily temperature as covariate, and area, start month, and carcass species as random factors, we found a positive correlation between scavenger diversity and carcass depletion time (Figure 5a; LMM, *df* = 42.533, *F* = 11.408, *p* = .002). Carcass initial state (LMM, *df* = 42.572, *F* = 0.192, *p* = .664) and mean daily temperature (LMM, *df* = 22.178, *F* = 3.554, *p* = .073) were not significant.

Second, we tested whether the diversity was higher when more scavenger groups were present on carcasses with a linear mixed‐effect model with the diversity index as dependent variable, the carcass groups and carcass initial state as fixed factors, and the previously mentioned covariate and random factors. Although we found that most of the scavenger groups did not differ in the diversity index, we did find that the carcasses with all the scavenger groups—Mammals, Birds, Wild boar and Occasionals—present had higher Shannon's diversity index than the carcasses with only the group Mammals and the groups Mammals and Wild boar present, and that the carcasses with Mammals, Birds, and Occasionals present had higher Shannon's diversity index than carcasses with only Mammals present (Figure 5b; LMM, *df* = 23.437, *F* = 5.729, *p* < .001). Again, carcass initial state (LMM, *df* = 30.957, *F* = 0.298, *p* = 0.589) and mean daily temperature (LMM, *df* = 25.048, *F* = 0.302, *p* = .587) were not significant.

## 4 DISCUSSION

This study aimed to determine whether functional differences among vertebrate scavengers occur, and how the diversity within the scavenger community relates to carcass decomposition speed. It became clear that the 17 selected vertebrate scavenger species were benefited from the presence of carcasses in different ways (Figure 2a,b; Table 2). More specialistic bird and mammal species, for example, common raven and European polecat, mainly used the carcasses directly as a food source, while occasional scavengers, for example, great tit and song thrush, used the carcasses rather indirectly by feeding on insects that they found on or close to the carcass. Great tit in particular often collected hair from the carcasses, which they presumably use for nest lining (Ondrušová & Adamík, 2013).

Although the grazers—cattle and horse—and one occasional scavenger—roe deer—are commonly known as obligate herbivores (Duncan & Poppi, 2008; Rørvang et al., 2018), we observed them sometimes showing EAT behavior (Table 2). Cattle was observed eating on bones and skin, horse on skin and soft tissue, and roe deer on hairs and skin (Table 2). These are all superficial and thus easily accessible, tissues types, indicating that these obligate herbivores tend to only scavenge in a simple and casual way. Scavenging by mammalian herbivores is a well‐documented phenomenon (Dudley et al., 2016), that is presumably a way for these species to ingest nutrients, which are otherwise rare in their diet (Bazely, 1989). This might imply that these species can find some nutrients that are beneficial to them in the tissues they consumed, for example, sodium (Na), magnesium (Mg), phosphorus (P), and potassium (K) from the skin and hairs, and calcium (Ca) and P from the bones (Wenting et al., 2020).

Among the non‐herbivore vertebrate species, the two domestic species in our data—dog and cat—contributed to the decomposition process of some carcasses. Most of these animals may be owned by humans in the surroundings of the study sites. For the dogs, we observed that they all wore a dog collar. Although there were no visible indications that the cats were owned by humans, we assumed that the observed individuals were suburban or farm cats since we only observed them on carcasses that had the nearest farm or house within a radius of 800 meters, which is a distance that could easily fall within a cat's home range (e.g., Barratt, 1997; Meek, 2003). Therefore, we presumed that these species did not have a real need to consume carcasses. Like obligate herbivores, their scavenging behavior can be described as only a minor part in their total diet. For this reason, it is unlikely that the presence of these species would replace or overrule the role of other—probably more important—scavenger species in the community (Huijbers et al., 2015). Among the other specialized scavenger species, there were instances of direct competition between individuals for carcasses, which never occurred among the grazers or occasional scavengers, indicating that the carcasses were an important resource for these species.

The behavior exhibited by the vertebrate species was consistent between our study areas; however, we cannot conclude that the scavenging behavior of these species would be the same across regions. The wood mouse, for example, was characterized as an occasional scavenger with very little eating behavior in our study (Table 2), while Young et al. (2014) found carrion to be a major part of their diet in the United Kingdom. This is probably a reflection of the local resource availability as wood mouse prefers to forage on seeds but relies on carrion when their preferred food source is scarce (Montgomery & Montgomery, 1990; Young et al., 2014). As another example, carrion crow and European polecat were among the more prevalent scavengers—classified as scavenging bird and mammalian scavenger, respectively—in our study, while Selva et al. (2005) described these species as minor, occasional scavengers in Bialowieza Primeval Forest, Poland. Since all the selected scavenger species in our study are facultative scavengers, resource availability presumably played an important role in determining their level of scavenging behavior.

Olson et al. (2012) found that the exclusion of key scavenger species from a community resulted in a longer depletion process. Obligate scavengers as vultures are often considered as the strongest competitors in the terrestrial scavenger guild (Houston, 1979) that can increase interspecific competition (e.g., Sebastián‐González et al., 2013) and in turn speed up carcass decomposition speed (Cortés‐Avizanda et al., 2012; Hill et al., 2018; Ogada et al., 2012). Although such obligate scavengers were absent from our study system, we found wild boar to be the dominant scavenger, with its presence enhancing the carcass decomposition speed (Figure 4a). Wild boar belonged to the species with the highest intraspecific interaction behavior (Table 2), indicating their social nature (e.g., Dardaillon, 1988; Maselli et al., 2014; Sebastián‐González et al., 2021). These results do not suggest that interspecific interactions between wild boar and other species did not occur, but is presumably a limitation of the annotation protocol that we used since we only annotated the longest and second‐longest shown behavioral type. Thus, we cannot conclude that interspecific interactions were absent, but we can conclude that intraspecific interactions occur more frequently and for longer periods than interspecific interactions. Vultures are described as the most specialistic species, able to rapidly consume carcasses (e.g., Cortés‐Avizanda et al., 2014; Mateo‐Tomás et al., 2017), and potentially triple the carcass decomposition speed (Ogada et al., 2012). Although we found that Wild boar presence did enhance the decomposition speed (Figure 4a), we cannot conclude that wild boar had such a tremendous effect. Therefore, although we denote wild boar as the dominant scavenger species in our study system, we cannot argue that this species had a comparable effect in our study system as vultures may have elsewhere.

Contrary to our expectation that a higher scavenger diversity would result in faster carcass decomposition, our results showed the opposite (Figure 5a). Probably our results can be explained by the longer monitoring periods when carcasses are decomposed at slower rates, resulting in longer windows of opportunity for species to detect and utilize the carcasses (Baruzzi et al., 2018). Accordingly, we found a higher diversity for carcasses with more scavenger groups involved in their decomposition process correlated with slow decomposition speed (Figure 5b). This implies that a slower carcass decomposition process would promote biodiversity the most, especially when taking into account the invertebrate species (e.g., Barton & Evans, 2017), while fast carcass decomposition by only a few vertebrate scavenger species might more substantially promote other natural processes, for example, the nutrient cycle, which is a key natural process for ecosystem functioning (e.g., Ngai & Srivastava, 2006). We speculate that the existence of variety in both the carcass decomposition speed and the differences in scavenger diversity within an ecosystem would contribute most to biodiversity and overall ecosystem functioning simultaneously.

In conclusion, defining the most dominant scavenger species in an ecosystem, complemented with the scavenger specialists and occasional scavengers, provides more insights into the role that the scavenging process plays in the area, and how it would affect biodiversity and fundamental natural processes simultaneously.

## CONFLICT OF INTEREST

No actual or potential conflicts of interest are declared by the authors.

## AUTHOR CONTRIBUTION

**Elke Wenting:** Conceptualization (equal); Data curation (equal); Formal analysis (equal); Funding acquisition (equal); Investigation (equal); Methodology (equal); Project administration (equal); Resources (equal); Supervision (equal); Validation (equal); Visualization (equal); Writing – original draft (equal). **Salomé C. Y. Rinzema:** Conceptualization (equal); Data curation (equal); Formal analysis (equal); Investigation (equal); Methodology (equal); Validation (equal); Visualization (equal); Writing – original draft (equal). **Frank van Langevelde:** Conceptualization (equal); Project administration (equal); Resources (equal); Supervision (equal); Writing – original draft (equal).

## Supporting information

Table S1‐S3

## Data Availability

The complete dataset will be accessible through Figshare: https://doi.org/10.6084/m9.figshare.14864850.

## References

[ece38576-bib-0001] Barratt, D. G. (1997). Home range size, habitat utilisation and movement patterns of suburban and farm cats *Felis catus* . Ecography, 20, 271–280.

[ece38576-bib-0002] Barton, P. S. , Cunningham, S. A. , Lindenmayer, D. B. , & Manning, A. D. (2013). The role of carrion in maintaining biodiversity and ecological processes in terrestrial ecosystems. Oecologia, 171, 761–772. 10.1007/s00442-012-2460-3 23007807

[ece38576-bib-0003] Barton, P. S. , & Evans, E. J. (2017). Insect biodiversity meets ecosystem function: differential effects of habitat and insects on carrion decomposition. Ecological Entomology, 42, 364–374. 10.1111/een.12395

[ece38576-bib-0004] Baruzzi, C. , Mason, D. , Barton, B. , & Lashley, M. (2018). (Effects of increasing carrion biomass on food webs. Food Webs, 17, e00096. 10.1016/j.fooweb.2018.e00096

[ece38576-bib-0005] Bazely, D. R. (1989). Carnivorous herbivores: Mineral nutrition and the balanced diet. Trends in Ecology & Evolution, 4, 155–156. 10.1016/0169-5347(89)90115-8

[ece38576-bib-0006] Benbow, E. M. , Barton, P. S. , Ulyshen, M. D. , Beasley, J. C. , DeVault, T. L. , Strickland, M. S. , Tomberlin, J. K. , Jordan, H. R. , & Pechal, J. L. (2019). Necrobiome framework for bridging decomposition ecology of autotrophically and heterotrophically derived organic matter. Ecological Monographs, 89, e01331. 10.1002/ecm.1331

[ece38576-bib-0007] Cortés‐Avizanda, A. , Jovani, R. , Carrete, M. , & Donázar, J. A. (2012). Resource unpredictability promotes species diversity and coexistence in an avian scavenger guild: a field experiment. Ecology, 93, 2570–2579. 10.1890/12-0221.1 23431588

[ece38576-bib-0008] Cortés‐Avizanda, A. , Jovani, R. , Donázar, J. A. , & Grimm, V. (2014). Bird sky networks: How do avian scavengers use social information to find carrion? Ecology, 95, 1799–1808. 10.1890/13-0574.1 25163114

[ece38576-bib-0009] Dardaiillon, M. (1988). Wild boar social groupings and their seasonal changes in the Camargue, southern France. Zeitschrift Für Säugetierkunde, 53, 22–30.

[ece38576-bib-0010] DeValult, T. L. , Rhodes, O. E. Jr , & Shivik, J. A. (2003). Scavenging by vertebrates: behavioral, ecological, and evolutionary perspectives on an important energy transfer pathway in terrestrial ecosystems. Oikos, 102, 225–234.

[ece38576-bib-0011] Dudley, J. P. , Hang’Ombe, B. M. , Leendertz, F. H. , Dorward, L. J. , de Castro, J. , Subalusky, A. L. , & Clauss, M. (2016). Carnivory in the common hippopotamus *Hippopotamus amphibius*: Implications for the ecology and epidemiology of anthrax in African landscapes. Mammal Review, 46, 191–203.

[ece38576-bib-0012] Duncan, A. J. , & Poppi, D. P. (2008). Nutritional ecology of grazing and browsing ruminants. The Ecology of Browsing and Grazing. Springer.

[ece38576-bib-0013] Farwig, N. , Brandl, R. , Siemann, S. , Wiener, F. , & Müller, J. (2014). Decomposition rate of carrion is dependent on composition not abundance of the assemblages of insect scavengers. Oecologia, 175, 1291–1300. 10.1007/s00442-014-2974-y 24859425

[ece38576-bib-0014] Feddern, N. , Mitchell, E. A. , Amendt, J. , Szelecz, I. , & Seppey, C. V. (2019). Decomposition and insect colonization patterns of pig cadavers lying on forest soil and suspended above ground. Forensic Science, Medicine and Pathology, 15, 342–351. 10.1007/s12024-019-00121-6 31129910

[ece38576-bib-0015] Freeman, P. W. , & Lemen, C. A. (2008). Measuring bite force in small mammals with a piezo‐resistive sensor. Journal of Mammalogy, 89, 513–517. 10.1644/07-MAMM-A-101R.1

[ece38576-bib-0016] Griffin, J. N. , de la Haye, K. L. , Hawkins, S. J. , Thompson, R. C. , & Jenkins, S. R. (2008). Predator diversity and ecosystem functioning: Density modifies the effect of resource partitioning. Ecology, 89, 298–305. 10.1890/07-1220.1 18409418

[ece38576-bib-0017] Hill, J. E. , DeVault, T. L. , Beasley, J. C. , Rhodes, O. E. Jr , & Belant, J. L. (2018). Effects of vulture exclusions on carrion consumption by facultative scavengers. Ecology and Evolution, 8, 2518–2526. 29531672 10.1002/ece3.3840 PMC5838040

[ece38576-bib-0018] Hooper, D. U. , Chapin, F. S. , Ewel, J. J. , Hector, A. , Inchausti, P. , Lavorel, S. , Lawton, J. H. , Lodge, D. M. , Loreau, M. , Naeem, S. , Schmid, B. , Setälä, H. , Symstad, A. J. , Vandermeer, J. , & Wardle, D. A. (2005). Effects of biodiversity on ecosystem functioning: A consensus of current knowledge. Ecological Monographs, 75, 3–35. 10.1890/04-0922

[ece38576-bib-0019] Houston, D. C. (1979). The adaptations of scavengers. Srengeti, dynamics of an ecosystem. University of Chicago Press.

[ece38576-bib-0020] Huijbers, C. M. , Schlacher, T. A. , Schoeman, D. S. , Olds, A. D. , Weston, M. A. , & Connolly, R. M. (2015). Limited functional redundancy in vertebrate scavenger guilds fails to compensate for the loss of raptors from urbanized sandy beaches. Diversity and Distributions, 21, 55–63. 10.1111/ddi.12282

[ece38576-bib-0021] Inagaki, A. , Allen, M. L. , Maruyama, T. , Yamazaki, K. , Tochigi, K. , Naganuma, T. , & Koike, S. (2020). Vertebrate scavenger guild composition and utilization of carrion in an East Asian temperate forest. Ecology and Evolution, 10, 1223–1232. 10.1002/ece3.5976 32076509 PMC7029075

[ece38576-bib-0022] Jones, K. E. , Bielby, J. , Cardillo, M. , Fritz, S. A. , O'Dell, J. , Orme, C. D. L. , Safi, K. , Sechrest, W. , Boakes, E. H. , Carbone, C. , & Connolly, C. (2009). PanTHERIA: a species‐level database of life history, ecology, and geography of extant and recently extinct mammals. Ecology, 90, 2648.

[ece38576-bib-0023] Kassambara, A. , & Mundt, F. (2017). Package ‘factoextra’: Extract and visualize the results of multivariate data analyses. Available from https://CRAN.R‐project.org/package=factoextra

[ece38576-bib-0024] Kuznetsova, A. , Brockhoff, P. B. , & Christensen, R. H. (2017). lmerTest package: Tests in linear mixed effects models. Journal of Statistical Software, 82, 1–26.

[ece38576-bib-0025] Le, S. , Josse, J. , & Husson, F. (2008). FactoMineR: An R package for multivariate analysis. Journal of Statistical Software, 25, 1–18.

[ece38576-bib-0026] Maselli, V. , Rippa, D. , Russo, G. , Ligrone, R. , Soppelsa, O. , D’Aniello, B. , Raia, P. , & Fulgione, D. (2014). Wild boars’ social structure in the Mediterranean habitat. Italian Journal of Zoology, 81, 610–917. 10.1080/11250003.2014.953220

[ece38576-bib-0027] Mateo‐Tomás, P. , Olea, P. P. , Moleón, M. , Selva, N. , & Sánchez‐Zapata, J. A. (2017). Both rare and common species support ecosystem services in scavenger communities. Global Ecology and Biogeography, 26, 1459–1470. 10.1111/geb.12673

[ece38576-bib-0028] Meek, P. D. (2003). Home range of house cats *Felis catus* living within a National Park. Australian Mammalogy, 25, 51–60. 10.1071/AM03051

[ece38576-bib-0029] Montgomery, S. S. J. , & Montgomery, W. I. (1990). Intrapopulation variation in the diet of the wood mouse *Apodemus sylvaticus* . Journal of Zoology, 222, 641–651.

[ece38576-bib-0030] Moore, J. C. , Berlow, E. L. , Coleman, D. C. , de Ruiter, P. C. , Dong, Q. , Hastings, A. , Johnson, N. C. , McCann, K. S. , Melville, K. , Morin, P. J. , & Nadelhoffer, K. (2004). Detritus, trophic dynamics and biodiversity. Ecology Letters, 7, 584–600.

[ece38576-bib-0031] Ngai, J. T. , & Srivastava, D. S. (2006). Predators accelerate nutrient cycling in a bromeliad ecosystem. Science, 314, 963. 10.1126/science.1132598 17095695

[ece38576-bib-0032] Ogada, D. L. , Torchin, M. E. , Kinnaird, M. F. , & Ezenwa, V. O. (2012). Effects of vulture declines on facultative scavengers and potential implications for mammalian disease transmission. Conservation Biology, 26, 453–460. 10.1111/j.1523-1739.2012.01827.x 22443166

[ece38576-bib-0033] Oksanen, J. , Guillaume Blanchet, F. , Friendly, M. , Kindt, R. , Legendre, P. , McGlinn, D. , Minchin, P. R. , O'Hara, R. B. , Simpson, G. L. , Peter Solymos, M. , Stevens, H. H. , Szoecs, E. , & Wagner, H. (2020). vegan: community ecology package. Available from https://CRAN.R‐project.org/package=vegan

[ece38576-bib-0034] Olson, Z. H. , Beasley, J. C. , DeVault, T. L. , & Rhodes, O. E. (2012). Scavenger community response to the removal of a dominant scavenger. Oikos, 121, 77–84. 10.1111/j.1600-0706.2011.19771.x

[ece38576-bib-0035] Olson, Z. H. , Beasley, J. C. , & Rhodes, O. E. (2016). Carcass type affects local scavenger guilds more than habitat connectivity. PLoS One, 11, e0147798. 10.1371/journal.pone.0147798 26886299 PMC4757541

[ece38576-bib-0036] Ondrušová, K. , & Adamík, P. (2013). Characterizing the mammalian hair present in Great tit (Parus major) nests. Bird Study, 660, 428–431.

[ece38576-bib-0037] Parmenter, R. R. , & MacMahon, J. A. (2009). Carrion decomposition and nutrient cycling in a semiarid shrub‐steppe ecosystem. Ecological Monographs, 79, 637–661. 10.1890/08-0972.1

[ece38576-bib-0038] Pereira, L. M. , Owen‐Smith, N. , & Moleón, M. (2014). Facultative predation and scavenging by mammalian carnivores: seasonal, regional and intra‐guild comparisons. Mammal Review, 44, 44–55.

[ece38576-bib-0039] R Core Team (2020). R: A language and environment for statistical computing. R Foundation for Statistical Computing. https://www.R‐project.org/

[ece38576-bib-0040] Ray, R. , Seibold, H. , & Heurich, M. (2014). Invertebrates outcompete vertebrate facultative scavengers in simulated lynx kills in the Bavarian forest national park, Germany. Animal Biodiversity and Conservation, 37, 77–88. 10.32800/abc.2014.37.0077

[ece38576-bib-0041] Rørvang, M. V. , Christensen, J. W. , Ladewig, J. , & McLean, A. (2018). Social learning in horses – fact or function? Frontiers in Veterinary Science, 5, 1–8. 30238009 10.3389/fvets.2018.00212 PMC6135911

[ece38576-bib-0042] Royal Netherlands Meteorological Institute (KNMI) . (2021). Weerstations ‐ Dagwaarnemingen. Available from https://daggegevens.knmi.nl

[ece38576-bib-0043] Schoenly, K. , & Reid, W. (1983). Community structure of carrion arthropods in the Chihuahuan desert. Journal of Arid Environments, 6, 253–263. 10.1016/S0140-1963(18)31510-6

[ece38576-bib-0044] Sebastían‐González, E. , Morales‐Reyes, Z. , Botella, F. , NavesAlegre, L. , & Pérez‐García, J. M. , Mateo‐Tomás, P. , Olea, P. P. , Moleón, M. , Barbosa, J. M. , Hiraldo, F. , & Arrondo, E. (2021). Functional traits driving species role in the structure of terrestrial vertebrate scavenger networks. Ecology, 2021, e03519. 10.1002/ecy.3519 34449876

[ece38576-bib-0045] Sebastían‐González, E. , Morales‐Reyes, Z. , Botella, F. , Naves‐Alegre, L. , Pérez‐García, J. M. , Mateo‐Tomás, P. , Olea, P. P. , Moleón, M. , Barbosa, J. M. , Hiraldo, F. , Arrondo, E. , Donázar, J. A. , Cortés‐Avizanda, A. , Selva, N. , Lambertucci, S. A. , Bhattacharjee, A. , Brewer, A. L. , Abernethy, E. F. , Turner, K. L. , … Sánchez‐Zapata, J. A. (2020). Network structure of vertebrate scavenger assemblages at the global scale: drivers and ecosystem functioning implications. Ecography, 43, 1143–1155. 10.1111/ecog.05083

[ece38576-bib-0046] Sebastían‐González, E. , Sánchez‐Zapata, J. A. , Donázar, J. A. , Selva, N. , Cortés‐Avizanda, A. , Hiraldo, F. , Blázquez, M. , Botella, F. , & Moleón, M. (2013). Interactive effects of obligate scavengers and scavenger community richness on lagomorph carcass consumption patterns. Ibis, 155, 881–885. 10.1111/ibi.12079

[ece38576-bib-0047] Selva, N. , & Fortuna, M. A. (2007). The nested structure of a scavenger community. Proceedings of the Royal Society B: Biological Sciences, 274, 1101–1108. 10.1098/rspb.2006.0232 PMC2124470 17301021

[ece38576-bib-0048] Selva, N. , Jędrzejewska, B. , Jędrzejewski, W. , & Wajrak, A. (2005). Factors affecting carcass use by a guild of scavengers in European temperate woodland. Canadian Journal of Zoology, 83, 1590–1601. 10.1139/z05-158

[ece38576-bib-0049] Smith, J. B. , Laatsch, L. J. , & Beasley, J. C. (2017). Spatial complexity of carcass location influences vertebrate scavenger efficiency and species composition. Scientific Reports, 7, 1–8. 10.1038/s41598-017-10046-1 28860543 PMC5578956

[ece38576-bib-0050] Swift, M. J. , Heal, O. W. , Anderson, J. M. , & Anderson, J. M. (1979). Decomposition in terrestrial ecosystems. Blackwell, Oxford.

[ece38576-bib-0051] Wenting, E. , Siepel, H. , & Jansen, P. A. (2020). Stoichiometric variation within and between a terrestrial herbivorous and a semi‐aquatic carnivorous mammal. Journal of Trace Elements in Medicine and Biology, 61, 126622. 10.1016/j.jtemb.2020.126622 32693327

[ece38576-bib-0052] Wilson, E. E. , & Wolkovich, E. M. (2011). Scavenging: how carnivores and carrion structure communities. Trends in Ecology & Evolution, 26, 129–135. 10.1016/j.tree.2010.12.011 21295371

[ece38576-bib-0053] WUR and INBO . (2021). Agouti. Wageningen University & the Research Institute for Nature and Forest. Retrieved from https://www.agouti.eu/

[ece38576-bib-0054] Young, A. , Stillman, R. , Smith, M. J. , & Korstjens, A. H. (2014). An experimental study of vertebrate scavenging behavior in a Northwest European Woodland Context. Journal of Forensic Sciences, 59, 1333–1342. 10.1111/1556-4029.12468 24611615

