# Supplementary material for: Functional differences in scavenger communities and the speed of carcass decomposition
Source: Ecol Evol. 2022 Feb 22;12(2):e8576. doi: 10.1002/ece3.8576 (PMC8861590; doi:10.1002/ece3.8576)
Supplement: Supplementary file 1 — Table S1‐S3 [file ECE3-12-e8576-s001.pdf]

Supporting information; Table S1. Overview of the carcasses included in this study.

ID	INDIVIDUAL CARCASS CODE	AREA	CARCASS SPECIES	INITIAL STATE*	CARCASS PLACEMENT	CARCASS DEPLETION	DAYS TO DEPLETION	SCAVENGER GROUP CODES**	MEAN DAILY TEMP. (°C)	WEATHER STATION	START MONTH
**1**	1e_Hogerwaarddwarsweg_1	Markiezaat	Roe deer	O	30-9-2019 15:02	30-10-2019	29.374	MBO	12.25	Woensdrecht	93
**2**	Hamert_1_Werkschuur_1	De Hamert Estate	Roe deer	C	17-10-2012 11:00	5-11-2012	18.542	M	9.46	Arcen	10
**3**	Hamert_1_Werkschuur_2	De Hamert Estate	Roe deer	C	27-11-2012 15:26	3-1-2013	36.357	MBO	4.83	Arcen	11
**4**	Hamert_1_Werkschuur_3	De Hamert Estate	Sheep	O	15-3-2013 11:56	27-3-2013	11.503	MBO	1.77	Arcen	15
**5**	Hamert_1_Werkschuur_4	De Hamert Estate	Roe deer	C	3-4-2013 15:26	13-6-2013	70.357	MB	11.75	Arcen	16
**6**	Hamert_3_Werkschuur_1	De Hamert Estate	Roe deer	O	20-7-2015 9:14	29-9-2015	70.615	MBO	16.69	Arcen	43
**7**	Hamert_3_Werkschuur_2	De Hamert Estate	Roe deer	C	6-10-2015 8:18	3-11-2015	27.654	M	9.24	Arcen	46
**8**	Hamert_3_Werkschuur_3	De Hamert Estate	Badger	C	5-1-2016 13:07	15-1-2016	9.453	M	5.08	Arcen	49
**9**	Hamert_2_Heereven_1	De Hamert Estate	Roe deer	C	8-6-2013 10:35	2-7-2013	23.559	M	16.56	Arcen	18
**10**	Hamert_2_Heereven_2	De Hamert Estate	Roe deer	C	9-1-2014 15:47	1-2-2014	22.342	M	4.51	Arcen	25
**11**	Hamert_2_Heereven_3	De Hamert Estate	Roe deer	C	13-2-2014 9:56	11-5-2014	86.586	MBO	10.00	Arcen	26
**12**	Hamert_2_Heereven_4	De Hamert Estate	Roe deer	O	12-5-2014 9:12	31-5-2014	18.617	MB	14.09	Arcen	29
**13**	Hamert_2_Heereven_5	De Hamert Estate	Roe deer	O	13-6-2014 10:25	10-7-2014	26.566	M	16.44	Arcen	30
**14**	Hamert_2_Heereven_6	De Hamert Estate	Roe deer	C	6-11-2014 10:40	27-1-2015	81.556	MO	5.16	Arcen	35
**15**	Hamert_2_Heereven_7	De Hamert Estate	Roe deer	C	21-3-2015 12:12	15-4-2015	24.492	MB	7.84	Arcen	39
**16**	Hamert_2_Heereven_8	De Hamert Estate	Roe deer	O	17-4-2015 14:18	13-5-2015	25.404	MO	11.94	Arcen	40
**17**	Hamert_2_Heereven_9	De Hamert Estate	Roe deer	O	27-1-2016 8:17	8-4-2016	71.655	MBO	5.62	Arcen	49
**18**	Hamert_4_Bosrand_1	De Hamert Estate	Roe deer	C	28-9-2018 12:49	21-11-2018	53.466	MBO	10.21	Arcen	81
**19**	KempenBroek_Loozerheide _1	Grenspark Kempen~Broek	Roe deer	C	27-2-2018 10:00	3-3-2018	3.583	MBG	-3.32	Ell	74
**20**	KempenBroek_Loozerheide _2	Grenspark Kempen~Broek	Roe deer	C	20-3-2018 10:00	1-5-2018	41.583	MBOG	10.59	Ell	75
**21**	KempenBroek_Stramproy _1	Grenspark Kempen~Broek	Roe deer	C	22-2-2018 12:25	17-3-2018	22.483	MBWOG	2.74	Ell	74
**22**	Markiezaat_1_DeDuintjes_1	Markiezaat	Roe deer	C	19-2-2018 10:28	7-3-2018	15.564	MBG	0.29	Woensdrecht	74
**23**	Melickerheide2_1	Meinweg National Park	Wild boar	C	21-5-2012 15:10	6-6-2012	15.368	MBWO	16.76	Ell	5
**24**	Melickerheide2_2	Meinweg National Park	Roe deer	C	7-6-2012 10:31	26-6-2012	18.562	MW	15.38	Ell	6
**25**	Wijffelterbroek_Graus2_1	Grenspark Kempen~Broek	Roe deer	C	8-10-2015 15:52	27-11-2015	49.339	MBWO	9.28	Ell	46
**26**	Meinweg_1_Eikelerveld_1	Meinweg National Park	Roe deer	C	6-11-2013 16:54	19-11-2013	12.296	MW	6.53	Ell	23
**27**	Meinweg_1_Eikelerveld_2	Meinweg National Park	Roe deer	O	25-11-2013 14:29	2-12-2013	6.397	BW	5.21	Ell	23
**28**	Meinweg_1_Eikelerveld_3	Meinweg National Park	Badger	O	6-2-2014 14:19	18-2-2014	11.403	MW	6.60	Ell	26
**29**	Meinweg_2_Slenk_1	Meinweg National Park	Roe deer	O	14-1-2016 12:00	12-2-2016	28.5	MBO	5.08	Ell	49
**30**	Meinweg_2_Slenk_2	Meinweg National Park	Badger	O	12-2-2016 13:09	24-2-2016	11.452	MB	3.42	Ell	50
**31**	Valkenhorst_Wildviaduct_1	Valkenhorst Estate	Roe deer	C	9-8-2019 13:39	14-8-2019	4.431	W	17.70	Ell	92
**32**	Valkenhorst_Wildviaduct_2	Valkenhorst Estate	Roe deer	O	11-11-2019 11:23	20-11-2019	8.526	BW	4.21	Ell	95
**33**	Valkenhorst_Wildviaduct_3	Valkenhorst Estate	Roe deer	C	28-2-2020 14:43	13-3-2020	13.387	MWO	7.41	Ell	98

Supporting information; Table S1. Overview of the carcasses included in this study (continue).

ID	INDIVIDUAL CARCASS CODE	AREA	CARCASS SPECIES	INITIAL STATE*	CARCASS PLACEMENT	CARCASS DEPLETION	DAYS TO DEPLETION	SCAVENGER GROUP CODES**	MEAN DAILY TEMP. (°C)	WEATHHER STATION	START MONTH
**34**	Wijffelterbroek_Graus1_1	Grenspark Kempen~Broek	Wild boar	C	27-9-2013 12:53	1-1-2014	95.463	MBWO	8.27	Ell	21
**35**	Wijffelterbroek_Graus3_1	Grenspark Kempen~Broek	Badger	O	24-6-2014 15:15	6-11-2014	134.365	MO	15.98	Ell	30
**36**	Veluwezoom_1_1	Veluwezoom National Park	Wild boar	C	3-7-2020 10:22	18-8-2020	45.568	WO	18.94	Deelen	103
**37**	Veluwezoom_2_1	Veluwezoom National Park	Wild boar	C	3-7-2020 11:49	4-8-2020	31.508	MWO	16.75	Deelen	103
**38**	Veluwezoom_3_1	Veluwezoom National Park	Wild boar	C	3-7-2020 12:21	20-8-2020	47.485	MWOG	19.10	Deelen	103
**39**	Veluwezoom_4_1	Veluwezoom National Park	Wild boar	C	3-7-2020 14:22	20-7-2020	16.401	MWOG	16.01	Deelen	103
**40**	Veluwezoom_6_1	Veluwezoom National Park	Wild boar	C	5-8-2020 14:09	29-8-2020	23.41	MBW	21.46	Deelen	104
**41**	Veluwezoom_11_1	Veluwezoom National Park	Fallow deer	C	30-10-2020 10:04	5-11-2020	5.581	MBW	10.99	Deelen	106
**42**	Brunink_1	Overijssel	Roe deer	C	6-8-2019 13:52	25-1-2020	171.422	MBO	10.08	Hupsel	92
**43**	Brunink_2	Overijssel	Roe deer	C	8-2-2020 12:18	22-2-2020	13.488	M	7.31	Hupsel	98
**44**	Brunink_3	Overijssel	Roe deer	C	30-3-2020 14:04	22-5-2020	52.414	MBO	10.65	Hupsel	99
**45**	Hegeveld_1	Overijssel	Roe deer	C	3-9-2019 8:55	16-11-2019	73.628	MO	11.45	Hupsel	93
**46**	Aamsveen_1	Overijssel	Roe deer	C	12-5-2020 12:00	2-7-2020	50.5	MBWO	16.09	Hupsel	101
**47**	PlankenWambuis_4_1	Planken Wambuis	Red deer	C	15-10-2019 9:15	20-10-2019	4.615	MW	12.52	Deelen	94
**48**	PlankenWambuis_7_1	Planken Wambuis	Red deer	C	28-10-2019 16:30	16-11-2019	18.313	MBW	6.20	Deelen	94
**49**	PlankenWambuis_8_1	Planken Wambuis	Red deer	C	1-11-2019 11:28	12-11-2019	10.522	MBW	7.23	Deelen	95

* C = closed carcass; decomposition started in bloated stage. O = opened carcass; decomposition started in active decay.

** Scavenger groups involved in the decomposition process: M = Mammals; B = Birds; O = Occasionals; and W = Wild boar.

Supporting information; Table S2. Number of carcasses per combination of scavenger groups, with Grazers included (A); without Grazers (B); and the selected combinations (C).

**A)**		**B)**		**C)**	
**CODE***	**Count**	**CODE***	**Count**	**CODE***	**Count**
BW	2	BW	2	M	7
M	7	M	7	MB	6
MB	4	MB	6	MBO	11
MBG	2	MBO	11	MBW	4
MBO	10	MBW	4	MBWO	5
MBOG	1	MBWO	5	MO	4
MBW	4	MO	4	MW	4
MBWO	4	MW	4	MWO	4
MBWOG	1	MWO	4		
MO	4	W	1		
MW	4	WO	1		
MWO	2				
MWOG	2				
W	1				
WO	1				
TOTAL	49	TOTAL	49	TOTAL	45

* Scavenger groups involved in the decomposition process: G = Grazers; M = Mammals; B = Birds; O = Occasionals; and W = Wild boar.

Supporting information; Table S3. Overview of the scavenger species that were present per study area. X = with showing scavenging behaviour; + = without showing scavenging behaviour.

	**Enschede area**	**Planken Wambuis**	**Veluwezoom National Park**	**Markiezaat**	**De Hamert Estate**	**Valkenhorst Estate**	**Grenspark Kempen~Broek**	**Meinweg National Park**
B. taurus			+				X	
E. caballus				X				
A. sylvaticus	X		+			+		+
C. capreolus	X		+	+	+	+	+	X
P. major	X			X	X		X	
T. philomelos	X		X		+			
T. pilaris			X					
T. viscivorus			X					
B. buteo	X	X	X	X	X	X	X	X
C. corax		X	+		X			
C. corone				X	X		X	
C. lupus familias					X	+	+	
F. catus					X		X	
M. foina	X				X		X	X
M. putorius	X				X		X	+
V. vulpes	X	X	X	X	X		X	X
S. scrofa	X	X	X			X	X	X
